# Surprising sirtuin crosstalk in the heart

**DOI:** 10.18632/aging.100128

**Published:** 2010-03-17

**Authors:** Thaddeus T. Schug, Xiaoling Li

**Affiliations:** Laboratory of Signal Transduction, National Institute of Environmental Health Sciences, National Institutes of Health, Research Triangle Park, NC 27709, USA

**Keywords:** Sirtuins, insulin-like growth factor, cardiac hypertrophy

Cardiovascular disease is the leading cause of death in the United States, accounting for nearly one third of all mortalities [9]. While lifestyle modifications, drug development, and surgical technologies have increased the average life expectancy of humans, the risk and rates of heart disease continue to grow as the population ages. The process of aging causes unique physiological, histological, and biochemical changes in cardiac tissue. For example, aging cardiac myocytes are incapable of proliferation and cannot be reprogrammed transcriptionally in response to changes in workload [7]. Age-associated conditions such as buildup of reactive oxygen species, mechanical dysfunction, or other forms of trauma, have been linked to the development of hypertrophy and other cardiac pathologies [6,14]. Therefore, development of drugs that target age-dependent signaling pathways may provide promising therapeutic strategies for the treatment of heart disease.

## mIGF-1 protects the heart through crosstalk with SIRT1

A study by Vinciguerra et al. published in the January 2010 issue of Aging furnishes new and important information about the cellular mechanisms leading to the pathogenesis of heart failure [22]. They propose a cardioprotective link between locally acting insulin-like growth factor (mIGF-1) and the NAD+-dependent deacetylase SIRT1. Typically, it is thought that the highly conserved IGF-1 and sirtuin signaling pathways play antagonizing roles in mammalian physiology. IGF-1 acts primarily as a growth hormone and signaling factor. Mice lacking GH/IGF-I signaling and IGF-1 receptor heterozygous knockout mice have longer lifespans, and overexpression of a hormone known to inhibit insulin/IGF-1 signaling extends lifespan [5,18]. However, the complex structure of the Igf-1 gene gives rise to multiple peptide isoforms that have contrasting functions [21]. Notably, the mIGF-1 isoform, which is expressed at high levels in neonatal tissues and adult liver, promotes regenerative properties in damaged heart tissue [21].

SIRT1, the mammalian orthologue of yeast Sir2, is a highly conserved NAD+-dependant protein deacetylases that has emerged as an important regulator of aging and metabolic disease [2]. SIRT1 and its family members are reported to promote longevity in different model organisms, including yeast, worm and fly [2,13]. The mammalian SIRT1 protein is primarily nuclear, and its functions have been tied to metabolism, cell survival and stress response [4]. The full-body SIRT1 knockout mouse displays ventricular adult heart abnormalities [10], but a severe developmental phenotype, together with high neonatal mortality rates make use of it difficult to study the physiological role of SIRT1 in the adult heart. Interestingly, high levels of SIRT1 expression (>9-fold) in the heart causes hypertrophy, loss of cardiac function, and elevated apoptosis [1]. On the other hand, moderate overexpression (2.5 to 7-fold) of SIRT1 in transgenic mouse hearts protects against oxidative stress, and results in increased expression of antioxidants [1]. As well, SIRT1 expression is increased in the hypertrophic heart of rodents and monkeys, though its functional relevance remains unclear [19].

**Figure 1.**
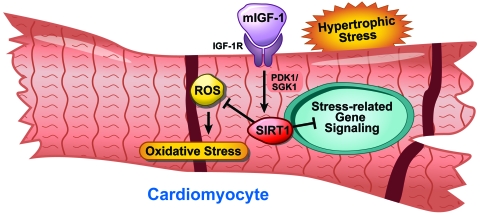
Model depicting the role of mIGF-1 regulation in cardiomyocytes.

Vinciguerra et al. hypothesize that although SIRT1 and circulating IGF-1 play opposite roles, the local mIGF-1 isoform displays a novel cross-talk signaling program with SIRT1, which results in cardiomyocite protection from hypertrophic and oxidative stress. They show that mIGF-1-mediated activation of SIRT1 induces expression of the protective signaling molecules UCP1, adiponectin, and MT2 [22]. They suggest that SIRT1 activation in the heart may also elicit protection from hypertrophy by restoring expression of fetal α-myosin heavy chain 7. The authors note an important distinction between circulating IGF-1 and mIGF-1. Typically, circulating IGF-1 activates PI3K/AKT/mTor and MAP kinase pathways, whereas mIGF-1 signals through PDK1 and SGK1 [22]. They conclude that the divergent signaling mechanisms between the two IGF-1 isoforms may account for their opposing effects in heart tissue (Figure 1).

**Table 1.** Mammalian Sirtuin cardiac phenotypes.


Sirtuin	Location	Activity	Transgenic Mouse Phenotype
SIRT1	Nucleus	NAD-dependent deacetylase	KO - Ventricular abnormalities
			Tg - Overexpression (>9-fold) - Cardiac hypertrophy
			Tg - Overexpression (2.5 to 7-fold) - Protection from oxidative stress - Increased expression of antioxidants
SIRT 3	Mitochondria/nucleus	NAD-dependent deacetylase	KO - Cardiac hypertrophy
			Tg - Overexpression - Cardio-protective
SIRT 7	Not established	NAD-dependent deacetylase/ ribosomal biogenesis	KO - Cardiac hypertrophy

## Other sirtuins benefit the heart

Recently, two additional sirtuin family members—SIRT3 and SIRT7--have been shown to have beneficial functions in the heart (Table 1), [17,20]. SIRT3, which consists of two isoforms (≈28 kDa and ≈44 kDa) was initially identified as a mitochondrial protein, but has since been identified in the nucleus as well [15,16]. Similar to SIRT1, SIRT3 uses NAD+ as a cofactor for the deacetylation of target substrates [2,12]. Cellular energy status, reflected in NAD+ levels and NAD+/NADH ratios, are thought to influence SIRT1 and SIRT3 expression and activity [8]. For example, mild stress conditions, such as mechanical stress to the heart and calorie restriction (CR), are presumed to reduce NADH levels, thus increasing the NAD+/NADH ratio. These alterations in cellular energy provide fuel to drive induction of SIRT3 during physiologic and mild hypertrophy.

SIRT3-deficient mice show signs of cardiac hypertrophy and interstitial fibrosis at 8 weeks of age, while SIRT3 transgenic overexpressing mice are protected from application of hypertrophic stimuli [17]. SIRT3 blocks the cardiac hypertrophic response through activation of Foxo-dependent antioxidants, manganese superoxide dismutase (MnSOD) and catalase, as well as suppressing ROS-mediated Ras activation and the downstream MAPK/ERK and PI3K/Akt signaling pathways [17]. SIRT1 and SIRT3 appear to share similar ROS-accumulating end-point targets that cause cardiac hypertrophy. Use and development of sirtuin-specific activators and inhibitors may help further dissect the collaborative functions of SIRT1 and SIRT3 in the heart.

Less is known about the physiological role of SIRT7 in the heart. SIRT7 is a nuclear protein that associates with rDNA and interacts with RNA [3]. It is not certain whether SIRT7 exhibits NAD+-dependent deacetylase activity, but reports suggest that it does respond to metabolic conditions by stimulating ribosomal biogenesis in dividing cells [11]. SIRT7-deficient mice develop heart hypertrophy and inflammatory cardio-myopathy, which is characterized by extensive fibrosis [20]. SIRT7 appears to regulate heart cell death and damage by inhibiting p53, Ras, and Akt signaling pathways [20]. The molecular details explaining how SIRT7 targets these pathways remains unclear.

## Conclusions

Sirtuins are longevity factors that also appear to regulate critical cardio-protective pathways in the mammalian heart. To date, three family members—SIRT1, SIRT3, and SIRT7—have been shown to block stress-induced cardiac hypertrophy by impinging upon ROS generation. It is interesting that knockout mice for each sirtuin isotype exhibit heart abnormalities, while transgenic overexpression of all three provides protection from cardiac hypertrophy. More investigation using conditional knockout models and specific activators is needed to elucidate the distinct molecular functions of each sirtuin. These studies will have profound implications, not only for the management of heart failure, but also for other stress-associated diseases.
